# Vertebral morphometrics and lung structure in non-avian dinosaurs

**DOI:** 10.1098/rsos.180983

**Published:** 2018-10-24

**Authors:** Robert J. Brocklehurst, Emma R. Schachner, William I. Sellers

**Affiliations:** 1 School of Earth and Environmental Sciences, University of Manchester, Manchester, UK; 2 Department of Cell Biology and Anatomy, School of Medicine, Louisiana State University Health Sciences Center, New Orleans, LA 70112, USA

**Keywords:** lung morphology, respiration, archosauria, axial skeleton, dinosauriformes

## Abstract

The lung-air sac system of modern birds is unique among vertebrates. However, debate surrounds whether an avian-style lung is restricted to birds or first appeared in their dinosaurian ancestors, as common osteological correlates for the respiratory system offer limited information on the lungs themselves. Here, we shed light on these issues by using axial morphology as a direct osteological correlate of lung structure, and quantifying vertebral shape using geometric morphometrics in birds, crocodilians and a wide range of dinosaurian taxa. Although fully avian lungs were a rather late innovation, we quantitatively show that non-avian dinosaurs and basal dinosauriforms possessed bird-like costovertebral joints and a furrowed thoracic ceiling. This would have immobilized the lung's dorsal surface, a structural prerequisite for a thinned blood-gas barrier and increased gas exchange potential. This could have permitted high levels of aerobic and metabolic activity in dinosaurs, even in the hypoxic conditions of the Mesozoic, contributing to their successful radiation.

## 1. Introduction

The respiratory system of non-avian dinosaurs has been the topic of considerable study over the years, both in an attempt to shed light on the biology of now extinct members of the Dinosauria, and in order to understand the origins and evolution of the highly derived lung-air sac system of modern birds [1–7]. Unfortunately, reconstructions of the dinosaur respiratory system based on phylogenetic bracketing are problematic, due to the disparity in lung morphology, physiology and ventilatory mechanics of their closest living relatives, birds and crocodilians [8–18]. Therefore, additional information from osteological correlates is needed.

Postcranial skeletal pneumaticity (PSP: invasion of the bone by respiratory diverticula), is an osteological correlate commonly used to reconstruct the presence of air sacs [3,6,19–25], but this only provides indirect information about the structure of the lungs themselves. As the lungs are the primary site of gas exchange in terrestrial vertebrates [26,27], reconstructing their anatomy and structure is essential for estimating key physiological parameters in fossil organisms [2,28]. The anatomy of the vertebrae, ribs and costovertebral joints has been proposed as an osteological correlate of lung structure [4,5]. In extant sauropsids, the dorsal surface of the lungs is directly adjacent to—and often attaches to—the vertebral bodies and rib heads. The dorsal region of the lung often has a denser concentration of gas-exchanging parenchyma, and a strong attachment to the body wall helps to prevent collapse [29–31]. Although overall axial morphology is also correlated with posture and locomotion [32,33], the anatomy of the costovertebral joint specifically relates directly to the gross morphology of the dorsal surface lung, and can be used as an osteological correlate to reconstruct lung structure in fossil taxa [4,5].

**Figure 1.**
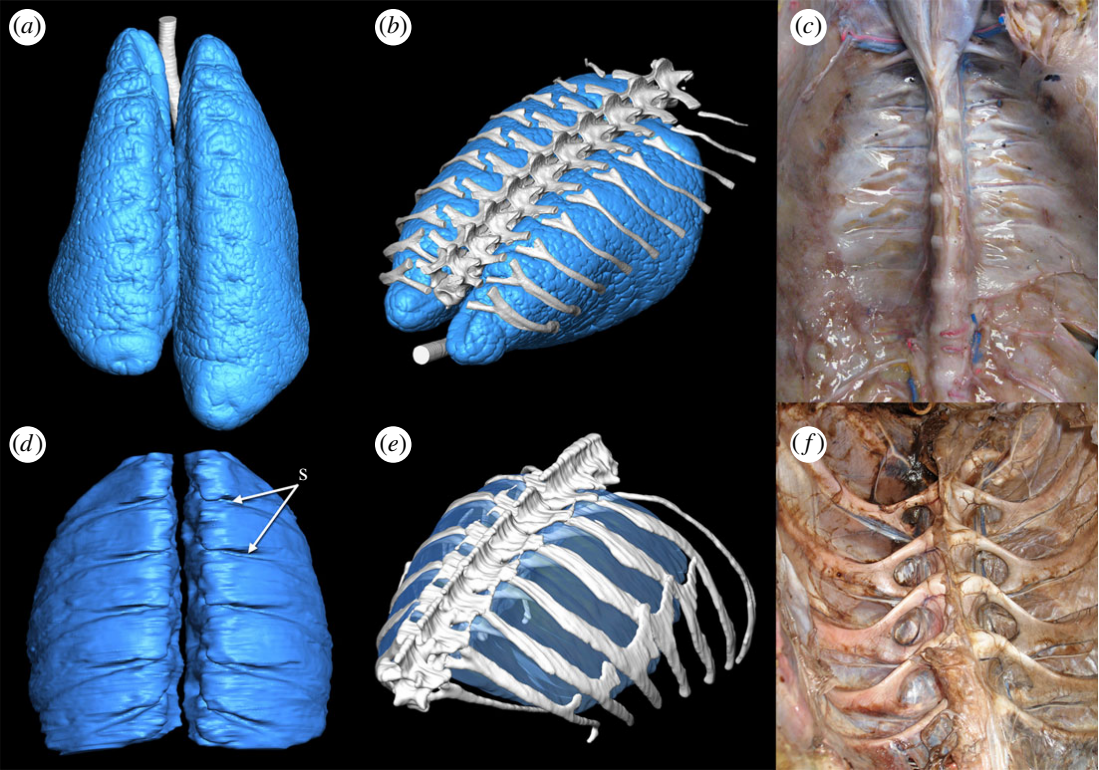
Anatomy of the lung and thorax of extant archosaurs. (*a*) Dorsal view of the lungs and trachea of a hatchling American alligator (*Alligator mississippiensis*) generated from microCT*.* (*b*) Lungs of a hatchling *A. mississippiensis* in association with the vertebral column and dorsal ribs in left anterolateral view. (*c*) Interior of the thoracic cavity of *A. mississippiensis* with all viscera removed. (*d*) Dorsal view of the gas-exchanging lungs of the African grey parrot (*Psittacus erithacus*) (no air sacs are shown). (*e*) Lungs of *P. erithacus* in association with the vertebral column and dorsal ribs in left anterolateral view. (*f*) Interior of the thoracic cavity of the ostrich (*Struthio camelus*) with all viscera removed. Segmented surface models in (*a*,*b*,*d*,*e*) generated in the visualization programme Avizo 7.1 from microCT DICOM data of inflated lungs *in situ*. Abbreviation: s, costal sulci. Images not to scale.

Both birds and crocodilians share bicapitate ribs, with two articulations to the vertebral column at the costovertebral joint—the rib capitulum with the vertebral parapophysis and the rib tuberculum with the vertebral diapophysis [34,35]. Differences in the anatomy of the joint between birds and crocodilians are associated with differences in the structure and mechanics of the lungs. In crocodilians, the anterior-most thoracic vertebrae have the parapophyses located on the vertebral centra, and the diapophyses are positioned on the distal ends of the transverse processes. From the third thoracic vertebra, the parapophysis begins to migrate onto the transverse process towards the diapophysis, and eventually the two articulations fuse and disappear [15]. This shift in the position of the parapophysis results in a reduction of the tuberculum, and combined with the broad and thin transverse processes, provides a smooth thoracic ceiling (figure 1) [4,5], which may facilitate antero-posterior motion of the viscera and mobile, compliant lungs during ventilation via the hepatic piston mechanism [5,15,36].

In birds, the parapophysis is located on the centrum for the entire dorsal series, and so the ribs are all strongly forked with a distinct capitulum and tuberculum. This creates a furrowed thoracic ceiling, where the dorsal surface of the lungs is deeply incised by the rib capitulae (figure 1); 20–33% of the lung tissue may lie between the rib heads [8,37]. Further, this immobilizes the lung's dorsal surface providing structural support for the lung as a whole, which experiences very little change in volume during breathing [38] and is practically rigid overall [8,13]. This immobility is considered a prerequisite for the extreme subdivision of the parabronchi and thinning of the blood-gas barrier [13,39,40].

Rib and vertebral morphology have been thoroughly described in dinosauriforms [4,5], but these descriptions are purely qualitative in nature. This lack of quantitative data means that currently, it is difficult to rigorously test hypotheses on the respiratory morphology of fossil archosaurs based on differences in the axial skeleton, particularly those that are subtle, or divergent from the avian or crocodilian anatomy. Additionally, a quantitative approach should yield finer detail in the differences between specific fossil groups, allowing for a clearer analysis of the progression of the evolution of the respiratory system along the avian stem. Based upon the anatomy observed in extant taxa, extinct individuals with a more bird-like ‘furrowed' thoracic ceiling can be parsimoniously reconstructed as having a stronger attachment between the lungs and the axial skeleton, which would be indicative of a more heterogeneous distribution of gas-exchanging parenchyma, with a densely partitioned dorsal region, and a more sac-like ventral region [5,29,31].

**Figure 2.**
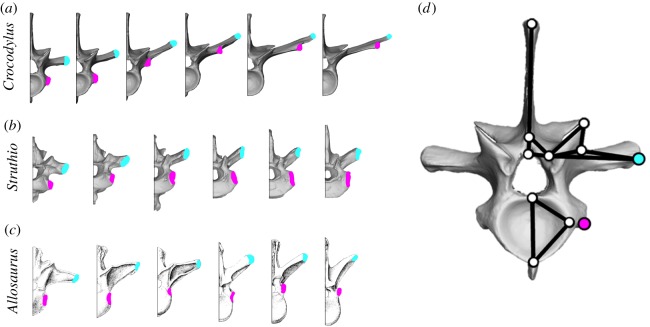
The diapophysis, parapophysis and other vertebral landmarks. (*a*–*c*) The six anterior-most dorsal vertebrae of (*a*) the extant crocodilian *Crocodylus americanus* (UMZC R6062), (*b*) the extant bird *Struthio camelus* (NMS 1879.85.9) and (*c*) the extinct theropod *Allosaurus fragilis* (Madsen, 1976 [41]) showing the positions of the parapophysis (pink) and the diapophysis (blue). (*d*) Landmarks used to quantify vertebral shape variation in archosaurs, shown on the first dorsal vertebra of *Crocodylus americanus* (UMZC R6062). For detailed descriptions of each landmark, see the electronic supplementary material.

In order to address these issues, we conducted a geometric morphometric analysis of dorsal vertebral morphology (figure 2) in a range of archosaur taxa, including extant birds and crocodilians, as well as fossil dinosauriforms. This allows us to test the hypothesis that basal dinosauriforms had vertebral (and hence, lung) morphologies intermediate between birds and crocodilians as proposed by Schachner *et al.* [4,5]. We also hypothesize that there is a phylogenetic progression towards a more ‘bird-like' condition, in terms of the structure and mechanics of the respiratory system (as evidenced by the morphology of the axial skeleton) beginning with basal dinosauriforms to crown group Aves. Ultimately this information should enable us to further refine our understanding of the morphology of the extinct dinosauriform lung, and the progression towards the fully avian-like respiratory system, with an immobilized gas-exchanging lung, and completely decoupled flexible ventilator air sacs.

## 2. Material and methods

Descriptions of the skeletal and pulmonary anatomy of birds and crocodilians are widely available in the literature [4,7,8,10,35,42–44]. For the lung models in figure 1, CT data were obtained from one hatchling *Alligator mississippiensis* and one *Psittacus erithacus.* The hatchling *Alligator* specimen was harvested from the Rockerfeller Wildlife Sanctuary for purposes unrelated to this study. Both the hatchling *Alligator* and *Psittacus* specimens had the trachea cannulated, inflated with a syringe and sealed. The thorax was scanned with the lungs inflated, using a Scanco µCT 40 at the Louisiana State University School of Veterinary Medicine (0.036–0.097 mm; 55 kVp 145 μA). The three-dimensional (3D) digital models were segmented with a Wacom Intuos Pro pen tablet in the scientific visualization programme Avizo v. 7.1 (https://www.fei.com/software/amira-avizo/).

For the morphometric analyses, the complete dorsal vertebral series of selected crocodilian and bird taxa was digitized either via surface scanning or computed tomography (CT) scanning. CT data were obtained for one subadult *Alligator mississippiensis*, which was scanned previously as part of an unrelated study, and one *Caiman crocodilus*. The *Caiman* specimen was intubated with plastic tubing, the lungs were artificially inflated via a 60 cc syringe and sealed with a stopcock prior to scanning. Scanning took place at the University of Utah Medical Center in South Jordan using a 164 slice dual energy Siemens SOMATOM Definition CT unit (slice thickness: 0.6–1 mm; 120 kVp 120, 200 mA). Three-dimensional digital models of the vertebrae were segmented in Avizo 8.0. All other specimens were surface scanned using a HP David SL3 structured light scanner. Individual scans were aligned and merged in David Software v. 5.0, to produce three-dimensional models of each vertebra. The extant dataset consists of the complete (or near-complete) dorsal series of four crocodilian taxa and 29 non-passerine birds, with a range of locomotor modes and body-forms. For a complete list of extant taxa used (see the electronic supplementary material, Methods).

For the fossil taxa, measurements were based on images and figures taken from the literature, as well as some observations of original material in museum collections. Vertebral series were sampled from Theropoda (four taxa), Sauropoda (three taxa) and the three main clades of Ornithischia (Thyreophora, Marginocephalia and Ornithopoda) (eight taxa) [5], as well as one non-dinosaurian dinosauriform, *Silesaurus opolensis* [45,46]. Taxa were chosen based on whether or not individual vertebrae could be seen in the required anterior view required for landmarking, and with ideally complete dorsal vertebral series. For a full list of the fossil taxa used in this study, as well as the appropriate references (see the electronic supplementary material, Methods).

Morphological variation of the dorsal vertebrae was quantified based on two-dimensional landmarks placed on vertebrae in anterior view (figure 2). Although three-dimensional analyses are increasingly common, two-dimensional analyses may be more applicable to fossil data which are often crushed or flattened [47], and overall shape variation between species captured by the landmarks is almost always greater than the discrepancies introduced by using two-dimensional morphometrics to represent three-dimensional structures [48]. As outlined above, the taxonomic sample covered a range of major clades of crocodilians, birds and non-avian dinosaurs, as well as various different body forms and locomotor styles within birds. In anterior view, the centrum, neural arch, parapophysis and diapophysis are all visible and identifiable using landmarks, which are homologous across archosaurs. As the vertebrae in this sample are approximately bilaterally symmetric, only the left side of each vertebra was digitized. If the left side was damaged or missing, specimens were mirrored as appropriate.

The landmark scheme used here is based on Head & Polly [47], with some modifications based on Böhmer *et al.* [49]; the 11 landmarks capture the height and width of the centrum, the height of the neural arch and neural spine, size and position of the prezygapophyses and the placement of the diapophysis and parapophysis (figure 2); the position of the parapophysis and diapophysis serving as our proxy for the ‘furrowedness’ of the thoracic ceiling. Detailed descriptions of each landmark are provided in the electronic supplementary material, Methods.

Landmarks were collected using *tpsDIG 32* [50], and imported into R [51], where they were analysed using *geomorph* [52]. Landmark data were superimposed using a Procrustes alignment to remove the effects of size, translation and orientation. Significance of differences between mean shapes for each major taxonomic grouping was assessed via a Procrustes ANOVA. A principal components analysis (PCA) was carried out for initial data visualization, and shape graphs showing the positive and negative extremes of the PC axes were also generated in *geomorph* [52]. Landmark data, in the form of .txt files, are available in the electronic supplementary material.

As well as the PCA, a linear discriminant analysis (LDA) was carried out in R using the MASS package [53]. This uses additional *a priori* information, increasing the chances of finding differences between groups. The extant dataset was split into three groups based on the descriptions of Schachner *et al*. [4,5] (i) birds, which all have a furrowed thoracic ceiling, forked ribs and the parapophysis on the vertebral centrum, (ii) anterior crocodilian vertebrae which create a furrowed thoracic ceiling, have forked ribs, and where the parapophysis lies on the vertebral centrum, and (iii) more posterior crocodilian vertebrae which create a smooth thoracic ceiling, with unforked ribs and where the parapophysis has migrated onto the transverse process. This LDA function was then used to classify fossil taxa into one of these three categories. When predicting the classifications of fossils, posterior probabilities for each assignment were generated using ‘leave-one-out' cross-validation in order to test the statistical robustness of each classification. The linear discriminant scores for both extant and extinct taxa were used to make morphospace plots similar to those generated by the PCA.

The linear discriminant scores from LD1, which separated vertebrae based on a smooth versus furrowed thoracic ceiling, were then used as a quantitative measure of the strength of rib forking (which in turn served as a proxy for dorsal lung immobility, and gross lung surface structure). LD1 scores were plotted against vertebral position to visualize changes in costovertebral joint anatomy along the vertebral column. Finally, an informal super-tree was assembled [54–56], and within-column variation in LD1 scores was compared across different phylogenetic groups in order to visualize when a furrowed thoracic ceiling appeared in the evolution of archosaurs.

## 3. Results

### 3.1. Measuring vertebral morphology

**Figure 3.**
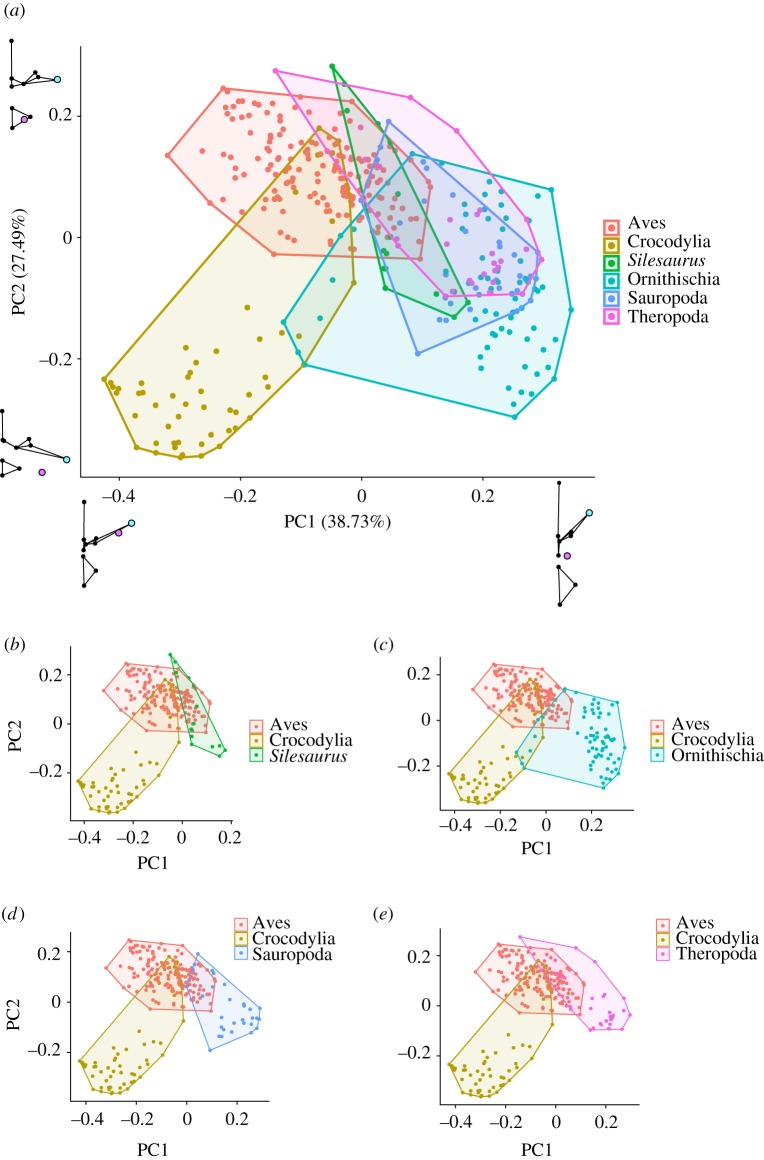
Principal components analysis. (*a*) Vertebral morphospace produced by PCA of the entire dataset. Shape graphs represent extremes of each PC axis. (*b*–*e*) Vertebrae from individual fossil groups compared with extant groups. Each point represents one vertebra. Taxa colour coded by taxonomic group.

A Procrustes ANOVA showed significant differences between the mean vertebral shapes of all major taxonomic groups (n_Aves_ = 164, n_Crocodylia_ = 57, n*_Silesaurus_* = 16, n_Ornithischia_ = 69, n_Sauropoda_ = 30, n_Theropoda_ = 30; *F* = 64.497; *p* < 0.05 in all cases) (electronic supplementary material, table S1). When examining effect sizes, fossil taxa were generally more similar to birds than to crocodilians, although ornithischians were equally distant from both (electronic supplementary material, table S1). A vertebral morphospace plot, derived from a principal components analysis (PCA), including the whole dataset, is presented in figure 3. The first PC axis accounts for 38.73% of the variation in the sample and roughly separates dinosaurs, particularly ornithischians, from extant archosaurs. High values of PC1 are associated with a relatively narrow centrum, proximally positioned parapophysis and short transverse process sharply angled to the horizontal. Conversely, lower values of PC1 show wide centra, distally placed parapophyses, and long, flat transverse processes. PC2, on other hand, accounts for 27.5% of the variation in the sample and seems to distinguish vertebrae which create a furrowed thoracic ceiling—birds, some dinosaurs and the anterior-most vertebrae in crocodilians—from those which create a smooth thoracic ceiling—crocodilian mid- and posterior-thoracic and lumbar vertebrae (figure 3). High values of PC2 are associated with a parapophysis located on the centrum and short transverse processes. Conversely, lower values of PC2 have the parapophysis located on the transverse process, as well as long transverse processes (figure 3).

### 3.2. Measuring costovertebral joint positioning

A second Procrustes ANOVA showed significant differences between the mean shapes for each of the three categories used in the LDA (n_bird_furrowed_ = 164, n_croc_furrowed_ = 10, n_croc_smooth_ = 47; bird_furrowed–croc_furrowed, *p* = 0.018; bird_furrowed–croc_smooth, *p* = 0.002; croc_furrowed–croc_smooth, *p* = 0.02). However, there was a much smaller effect size between the two furrowed groups than between either of these and the smooth group (bird_furrowed–croc_furrowed, *Z* = 2.69164; bird_furrowed–croc_smooth, *Z* = 17.30133; croc_furrowed–croc_smooth, *Z* = 10.41833).

The LDA was generally successful at assigning correct categories to the extant dataset. Bird vertebrae were correctly classified 100% of the time, and posterior crocodilian vertebrae, which created a smooth thoracic ceiling, were correctly classified approximately 98% of the time. Anterior crocodilian vertebrae, which created a furrowed thoracic ceiling, were classified correctly 70% of the time. Almost all dinosauriform vertebrae were classified along with either birds or crocodilian vertebrae with the parapophysis on the centrum, with high posterior probabilities. The full results from the LDA are available in electronic supplementary material, table S2.

**Figure 4.**
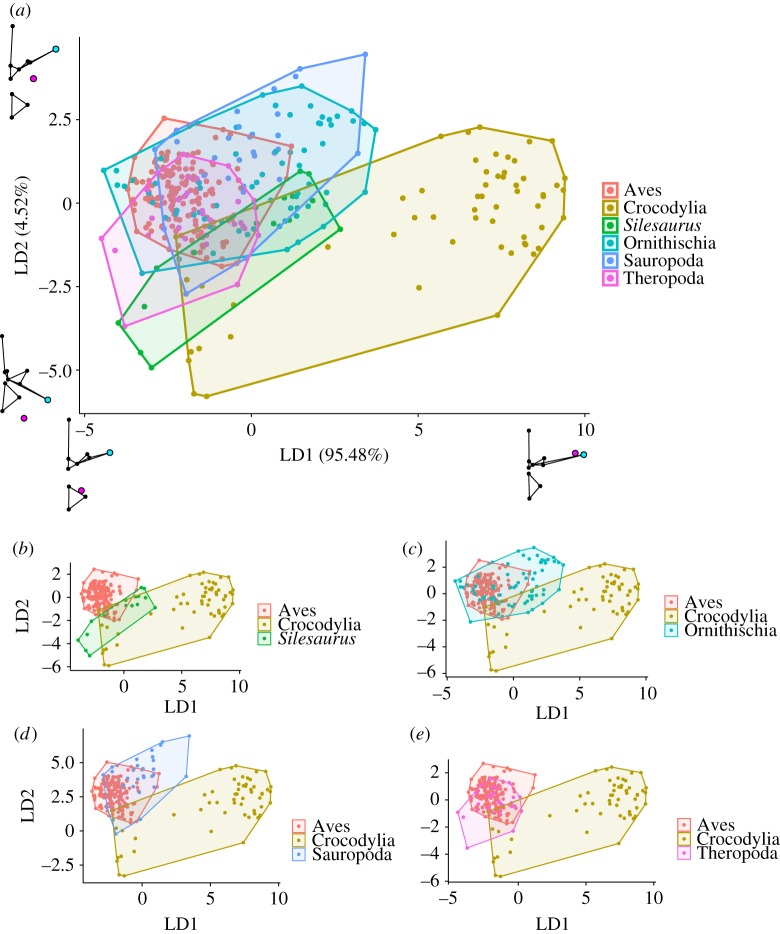
Linear discriminant analysis. (*a*) Vertebral morphospace produced using LDA. Linear discriminant scores are generated using the extant dataset; then, the linear discriminant scores are used to estimate the position of fossil taxa in this space. Shape graphs represent extremes of each LD axis. (*b*–*e*) Vertebrae from individual fossil groups compared with extant groups. Each point represents one vertebra. Taxa colour coded by taxonomic group.

In the morphospace based on the linear discriminant scores from the LDA, LD1 explained 95% of the shape variation between groups (figure 4). This was effectively an axis of how far the diapophysis and parapophysis were separated and how furrowed or smooth the thoracic ceiling was, combining elements of PCs 1 and 2 from the PCA (figures 3 and 4). LD1 was dominated by the position of the parapophysis relative to the diapophysis, as well as the length of the transverse processes; low values of LD1 are associated with a parapophysis positioned on the centrum, and short transverse processes. High scores for LD1, on the other hand, are associated with a parapophysis located on the transverse process, close to the diapophysis and long transverse processes (figure 4). LD2 accounted for the remaining 5% of the variation, and is associated with the morphology of the prezygopophyses, neural arch and angle of the transverse processes to the horizontal.

### 3.3. Intra-columnar variation in the thoracic ceiling

**Figure 5.**
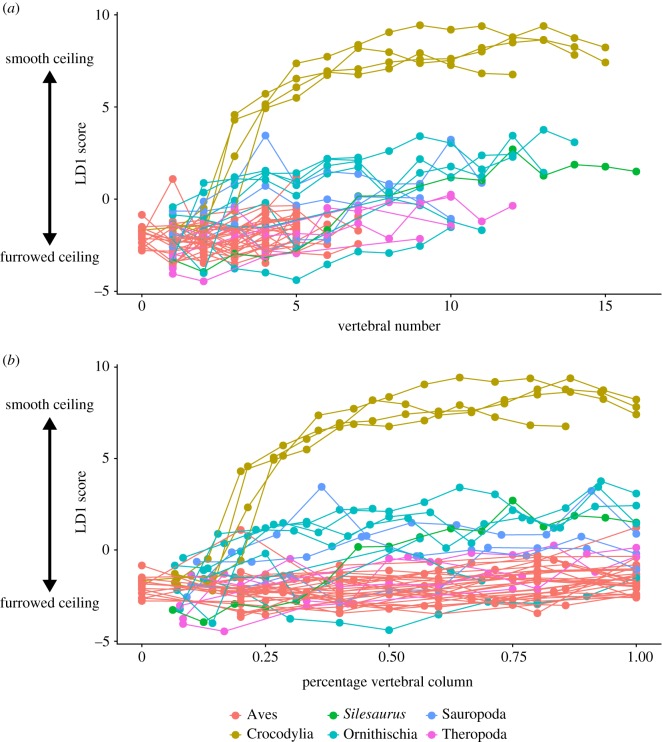
Shape variation along the vertebral column. (*a*) Vertebral number plotted against the linear discriminant 1 score. (*b*) Same, but with vertebral number normalized according to the total number of dorsal vertebrae in each taxon. Taxa colour coded by taxonomic group.

Variation in smoothness of the thoracic ceiling along the vertebral column (represented by the LD1 score) is shown in figure 5. Birds retain low values (i.e. furrowed thoracic ceiling) throughout the dorsal series. In crocodilians, LD1 values are low in the anterior-most vertebrae, but then there is a sharp increase in LD1 values and the thoracic ceiling becomes substantially smoother at the 3rd/4th dorsal vertebra, corresponding to 20–25% along the dorsal series in these taxa. In most dinosauriform taxa, the thoracic ceiling is furrowed anteriorly (low values along LD1), and although there is a general trend towards increased LD1 scores posteriorly, none of the fossil taxa reach the same very high LD1 values seen in crocodilians.

### 3.4. Evolutionary variation in the thoracic ceiling

**Figure 6.**
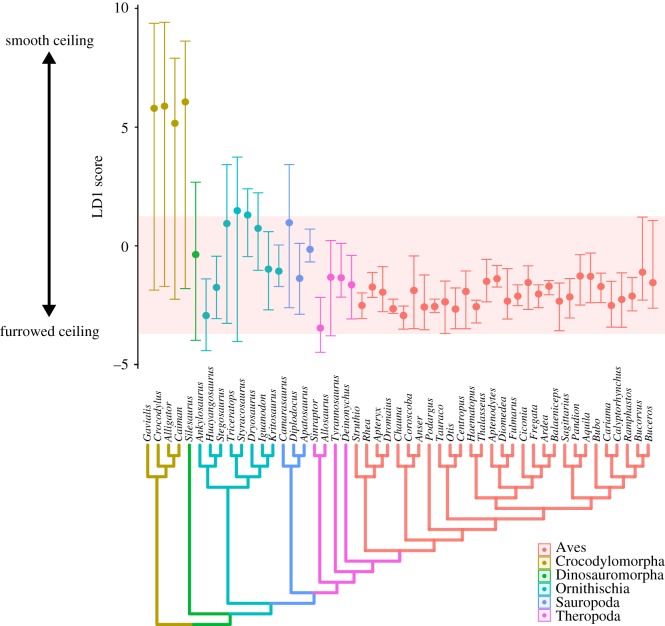
Shape variation across phylogeny. An informal supertree of the taxa used in this study, with the range and mean of the linear discriminant 1 scores for the whole vertebral column plotted for each taxon in the phylogeny. Taxa colour coded by taxonomic group. The red band represents the range of values seen in extant birds.

Phylogenetic variability in vertebral morphology and costovertebral joint anatomy—again, represented by LD1 score—is shown in figure 6. Crocodilians show the greatest range of morphological variation along the vertebral column, and their mean vertebral shapes have the highest scores along the first LD axis. Individual avian taxa show much less within-column variation (probably due in part to their lower dorsal vertebral counts), although there is still considerable variation between different genera. All birds have low mean LD1 scores. Many dinosauriform taxa fall within the range of variation seen in modern birds, particularly theropods, and all dinosauriforms have mean values below those of crocodilians.

## 4. Discussion

This analysis builds on previous qualitative studies on the archosaurian respiratory system and axial skeleton [4,5]. Our methods clearly separate extant birds and crocodilians as these two groups plot out distinctly in the PCA and the Procrustes ANOVA found significantly different group means. The LDA easily discriminated between vertebrae which produced a ‘furrowed' or ‘smooth' thoracic ceiling. Two main aspects of vertebral morphology, the length of the transverse processes and the relative positions of the diapophysis and parapophysis, have been previously identified as major correlates of lung structure and ventilation mechanics in archosaurs [4,5,15,16]. Both of these features strongly loaded the first two PC axes of the PCA (figure 3), as well as the first (and major) axis of the LDA (figure 4). Birds have short transverse processes and the parapophysis remains on the centrum, creating a furrowed thoracic ceiling in articulation with the ribs, which have a distinct capitulum and tuberculum (associated with the ventral parapophysis). In crocodilians, the anterior vertebrae are similar to birds—short transverse processes and parapophysis on the centrum—and indeed, this resulted in some overlap between birds and crocodilians in the PCA (figure 3). However, in the more posterior vertebrae, the parapophysis migrates onto the transverse processes and the transverse processes themselves are relatively long and flat (figures 3 and 4); combined with the reduction in the rib tuberculum associated with the parapophysis' migration, this creates a smooth thoracic ceiling.

By precisely quantifying the relative position of the costovertebral joint as an osteological correlate, we aimed to provide a more detailed, fine-scale comparison of the lung structure of different dinosauriform groups. However, we found limited evidence to support a phylogenetic progression towards an increasingly ‘furrowed' thoracic ceiling and a more bird-like lung structure, as all dinosaurian taxa showed osteological correlates of dorsally immobile lungs. Although the initial PCA and Procrustes ANOVA showed that fossil dinosauriforms occupied different regions of vertebral morphospace from extant groups, they are all still characterized by a distinct separation of the parapophysis and diapophysis—the parapophysis being placed ventral and substantially medial to the diapophysis, even if it has migrated away from the vertebral centrum. In line with this, most dinosauriform taxa plotted more closely towards the ‘furrowed' end of the first LD axis, and the LDA classed most dinosaurs as having vertebrae which would create a ‘furrowed' thoracic ceiling, with high posterior probabilities.

Despite gross morphological differences, extant birds and crocodilians do share many pulmonary characteristics, including major components of their bronchial tree, and patterns of airflow within the lung [17,44,57]. Based on extant phylogenetic bracketing, we might assume that fossil archosaurs had a heterogeneously partitioned lung, with a less mobile antero-dorsal region containing more of the respiratory parenchyma, and a more compliant, saccular ventral region, which probably served a primarily ventilatory role [7,17]. Extant phylogenetic bracketing also suggests that the lungs were supported by a post-pulmonary septum (PPS), with the body divided into a pleural and peritoneal cavity [58]. However, the results of our morphometric analysis indicate that the common ancestor of Dinosauria possessed a lung which was immobilized across the whole of the dorsal surface, implying more extreme partitioning and regionalization of gas exchange and ventilator functions. This also suggests the presence and increased role of an intracoelomic septum, which would support the saccular portion of the lung during ventilation and prevent collapse during exhalation [58].

Whether the more extreme partitioning of the respiratory system implied here in basal dinosauriforms represents an ancestral condition for Ornithodira, or maybe even for Archosauria, remains unclear. Pterosaurs, the ornithodiran outgroup to dinosauriforms, have been reconstructed as having an avian-style respiratory system based on the presence of unambiguous PSP and inferred air sacs [59,60]. Their costovertebral morphology has been described as ‘crocodilian-like', as the parapophysis migrates dorsally to lie on or at the base of the transverse process, and it has been suggested that pterosaurs therefore had more compliant, more homogeneous lungs [61]. However, the parapophysis also migrates dorsally towards the transverse process in many dinosaurian taxa, and our results showed greater similarity between dinosaurs and birds rather than dinosaurs and crocodilians. Further quantitative studies focusing on pterosaurs are clearly needed.

The sister taxon to Archosauria, the lepidosaurs, generally have compliant lungs, and are considered to have a ‘smooth' thoracic ceiling [5]. However, lepidosaurs also lack transverse processes, and the diapophysis and parapophysis are fused into a single joint, the synapophysis [5,34]. Additionally, the pulmonary anatomy and ventilator mechanics are extremely diverse across the various groups, with some having complex lungs that are dorsally attached to the body wall (e.g. varanids) and others possessing more simplified mobile lungs (e.g. teiids) [30,58]. Therefore, further osteological correlates need to be identified to investigate pulmonary evolution in lepidosaurs.

We now proceed to reconstruct the evolution of the respiratory system through the evolution of dinosauriforms. Beginning at the base of the tree, the reconstructions of *Silesaurus* [46] represent the most complete dorsal series known from a non-dinosaurian dinosauriform. Generally, *Silesaurus* vertebrae had low LD1 scores, with the more anterior vertebrae falling within the range of values seen in extant birds (figures 5 and 6). Only in the posterior half of the ribcage is there an increase in LD1 scores, but these are well below the values seen in the posterior ribcage of crocodilians (figure 5). All vertebrae in *Silesaurus* were classed as creating a ‘furrowed' thoracic ceiling by the LDA, with high posterior probabilities. The vertebral anatomy of *Silesaurus* combined with the morphology of the ribs, which possess a clearly separated tuberculum and capitulum [5,46], would have produced a furrowed thoracic ceiling. This, in turn, indicates that *Silesaurus* had a heterogeneously partitioned lung, with an immobile dorsal region that probably contained most of the gas exchange tissue and vasculature.

Previous descriptive work on the vertebral anatomy of ornithischians noted that they, of all the non-avian dinosaurs, most closely resembled crocodilians, particularly in the position of the parapophosis [5]. This character is variable among Ornithischia, as some taxa have the parapophysis on the neural arch, but in others it may be positioned at the base of the transverse process or on the transverse process itself [5]. The ornithischian vertebrae included in our sample had the parapophysis ventral to the diapophysis, and it never fully migrated to the distal end of transverse processes. As a result, in our analysis, most ornithischians had relatively low LD1 scores, and were generally classed as having ‘forked' ribs by the LDA. Nevertheless, certain ornithischian taxa were picked out by the LDA as showing crocodilian traits in their vertebral morphology; both *Stegosaurus* and *Triceratops* had vertebrae classified as creating a ‘smooth' thoracic ceiling. However, the LD1 scores were still much lower than those seen in crocodilians. In a variety of small-bodied ornithischian taxa, the diapophysis and parapophysis sometimes fuse into a single facet in the posterior-most dorsals [62–64]. However, even in taxa such as this, ornithischians retain distinct bicapitate dorsal ribs along all (or at least most) of their dorsal series, and so would also have had a furrowed thoracic ceiling. Ornithischians, therefore, generally show evidence of a dorsally immobile lung, and even the specific exceptions discussed above still show less dorsal mobility and a more furrowed thoracic ceiling than in extant crocodilians, especially when considering the morphology of both the vertebrae and ribs.

Both non-dinosaurian dinosauriforms and ornithischian dinosaurs lack unambiguous evidence of PSP and so it is difficult to make inferences about the non-gas-exchanging regions of the lung in these groups. However, Butler *et al.* [25] do raise the possibility of non-invasive respiratory diverticula, and possibly air sacs, being present in both *Silesaurus* and ornithischians, as an ancestral feature of Ornithodira. The cervical and anterior dorsal vertebrae of *Silesaurus*, as well as some ornithischians, possess strongly developed laminae and deep fossae [25,46] and there are similarities in the distribution and development of these ‘ambiguous' pneumatic features (i.e. features possibly associated with respiratory diverticula) and unambiguous features found in other taxa [25]. The ‘common pattern' of pneumaticity in extant birds has PSP limited to the cervical and anterior dorsal vertebrae [65], and this is also where the first unambiguous evidence of PSP occurs in pterosaurs, sauropods and theropods [6,25]. This provides support for the features in *Silesaurus* and ornithischians being potentially associated with respiratory diverticula, although this still requires further testing, e.g. using newly identified histological correlates for bone and air sac associations [66].

Descriptions of preserved sauropod ribcage elements show the separation of the diapophysis and parapophysis, and the consistently forked ribs [5]. This is supported by the results of this analysis, where the sauropod taxa examined had generally low scores on LD1, and fit broadly within the range of values seen in extant birds (figures 5 and 6); indeed, *Apatosaurus* and *Diplodocus* fit entirely within this range. Additionally, all sauropod vertebrae were classified as creating a ‘furrowed' thoracic ceiling in the LDA, apart from a few vertebrae in *Camarasaurus*. Basal sauropodomorphs (prosauropods) have been previously described as having a distinct parapophysis and diapophysis, as well as short transverse processes [5]; the parapophysis is on the neural arch in middle and posterior dorsals, and though it does not usually merge with the diapophysis, it can be positioned just anteroventral to it on the posterior-most dorsals. The first four ribs were strongly forked, and while this was followed by a gradual reduction in the tuberculum, all ribs remain bicapitate [5]. Therefore, the overall picture of respiratory evolution in both basal sauropodomorphs and sauropods is that they all possessed a well-separated diapophysis and parapophysis, bicapitate ribs, a furrowed thoracic ceiling, and the dorsal surfaces of the lung were immobilized by the adjacent ribs and vertebrae.

Theropods have been previously described as having the most ‘distinctly avian' axial morphology [4,5]. The taxa included in this study included one taxon from most of the main clades of Theropoda—Tetanurae, Coelurosauria and Maniraptora. There was no observable trend towards an increasingly avian vertebral structure moving crown-wards towards birds; however, more specimens from a broader phylogenetic range of taxa are required to properly evaluate any specific trends in this group. Theropods have a very separate parapophysis and diapophysis for the entire vertebral series [5], and showed low LD1 scores as a result (figures 4 and 5). All theropods had vertebrae classified as creating a ‘furrowed' thoracic ceiling in the LDA, and all taxa fell within the range of LD1 values seen in extant birds (figures 5 and 6). Although not included in this analysis, descriptions of more basal theropods, e.g. the abelisauroid *Majungatholus* note separate parapophysis and diapophysis, and ribs with a distinct capitulum and tuberculum [5,67]. These results support the reconstruction of a dorsally immobilized lung in non-avian theropods.

In both sauropods and theropods, the presence and distribution of PSP has been widely used to infer the presence of bird-like air sacs, and imply the presence of a bird-like lung [3,24,68,69]. Respiratory diverticula originating from specific air sacs pneumatize specific regions of the skeleton adjacent to the skeleton [20]. Whereas older accounts suggested that diverticula from the cervical air sacs and lungs can extend from the all the way down to the pelvic region [70], these findings could not be replicated [20]. Some juvenile birds exhibit a ‘pneumatic hiatus', or a gap in the pneumatization of the vertebral column indicative of an anterior and posterior centre of pneumatization [71,72]. Although absent in the most basal members of each clade [6,73], there are several examples of this, and similar ‘pneumatic diminutions' (a reduction in the extent of pneumatization), in the fossil record of both sauropodomorphs [24,69,73,74] and theropods [3,23,75]. This indicates that the diverticula responsible for pneumatizing the axial skeleton originated from multiple sources, further suggesting the presence of air sacs.

However, in addition to their associations with respiratory diverticula, avian air sacs have strict embryological and topological distinctions from the homologous ‘saccular regions' of the crocodilian lung [17]. In birds the air sacs are projections of the lung, separated from it by the horizontal and oblique septa, a relationship laid down early on in development when the air sac primordia invade the PPS, splitting it into two distinct septa [76,77]; by contrast, in other sauropsids, including crocodilians, lung development occurs along the pleural aspect of the PPS, so that the saccular regions are not projections from the rest of the lung, and the whole lung has a smooth outer contour [17]. Therefore, in order to say a fossil taxon truly possessed air sacs requires additional evidence of a bipartite PPS. The patterns and distribution of pneumaticity observed in fossils could also be explained by pulmonary diverticula emerging from the anterior and posterior saccular regions of a heterogeneous, ventrally flexible lung not bounded by horizontal and oblique septa. This would be a transitional stage towards the avian condition, but is not a fully decoupled exchanger and ventilator (i.e. immobilized lung and flexible air sacs) as seen in extant birds.

It is possible that some more derived theropods did have a fully decoupled lung and air sacs, with the immobile lung supported ventrally by a bipartite PPS, as in birds. This has been speculated based on the presence of well-developed hypapophyses in maniraptoran theropods [58]. The hypapophyses act as attachment sites for the median fibres of the horizontal and oblique septa [8], and so are a potential osteological correlate for the avian bipartite PPS. The presence of this bipartite PPS would have provided additional structural support for a rigid immobile lung, and physically separated the gas-exchanging regions from the air sacs, completing the division of the respiratory system into a functionally discrete exchanger and ventilator, as seen in extant birds [58]. This remains somewhat speculative, however, as the hypapophyses are also present in crocodilians, and in both birds and crocodilians serve as an attachment for the longus colli muscles [78,79].

It should also be noted in discussions of PSP that respiratory diverticula themselves have not been shown to serve any respiratory function, beyond providing evidence of air sac-like structures [80]. In extant birds, increased PSP beyond the cervicothoracic region is generally associated with increasing body mass [65]. In theropod dinosaurs, increased PSP has also been observed in large-bodied taxa [6] further suggesting its main role was in mass reduction. The prevalence of PSP in sauropods, which are almost all uniformly large [24,81], and pterosaurs which need to reduce mass for flight [59,60], also suggests a role in mass reduction.

**Figure 7.**
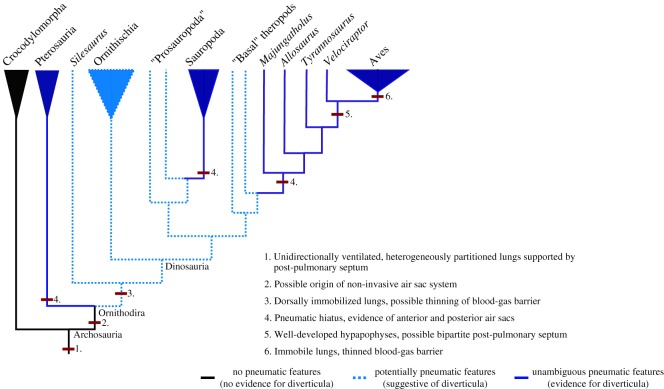
Evolution of the archosaur respiratory system. A phylogeny of Archosauria, showing key modifications to the respiratory system associated with the evolution of the avian lung-air sac system.

Therefore, we propose the following scenario for the evolution of the dinosauriform respiratory system (figure 7). A dorsally immobile lung, strongly partitioned into gas-exchanging and ventilatory regions, which may have been associated with non-invasive diverticula, was present in the immediate ancestors of dinosauriforms [25] (figure 7). In sauropods and theropods respiratory diverticula became invasive, associated with the presence of unambiguous PSP. This was presumably as a means of body mass reduction, as an adaptation to large body size [6,24] (figure 7). The distribution of PSP suggests that these taxa possessed anteriorly and posteriorly positioned flexible air sacs, or at least saccular regions of the lung. In more derived maniraptoran theropods, the body size threshold at which increased PSP evolved was reduced, possibly as an adaptation to high metabolic rates [6], and the presence of well-developed hypapophyses on the dorsal vertebrae may indicate the presence of an avian, bipartite PPS, and a fully decoupled immobilized lung and flexible air sacs [58] (figure 7).

Recently, major reorganizations have been proposed for the dinosaurian tree, with the traditional Saurischia–Ornithischia split rejected in favour of the ‘Ornithoscelida' hypothesis, where theropods and ornithischians are sister taxa, to the exclusion of sauropodomorphs [82,83]. While this has major implications for the evolution of some dinosaurian characters, it is unlikely to alter our conclusions on respiratory evolution a great deal. Our results support the presence of a bird-like, highly partitioned lung in all dinosaurs—and even non-dinosaurian dinosauriforms—before the three major clades of dinosaurs split from one another (figure 7). Also, although often thought of as a shared saurischian character, the evolution of unambiguous PSP and a pneumatic hiatus occurred independently in sauropods and theropods (figure 7).

The anatomy of the costovertebral joint in non-avian dinosaurs provides information not only on the structure of the lungs, but also on how they were potentially ventilated. The morphology of the vertebrae in crocodilians is suggested to correlate with hepatic piston breathing [4]. Experimental fluoroscopy data clearly show the anteroposterior motion of the viscera and the displacement of the lung's posterior border [15,36]; the smooth thoracic ceiling is hypothesized to facilitate this motion [4]. This is also associated with vertebrae with very high LD1 scores (figures 4–6). Birds, by contrast, have a furrowed thoracic ceiling which contributes to the immobility of the volume-constant avian lung, which is ventilated by compliant ‘extra-pulmonary' air sacs via costosternal pumping [5,7]. It has been previously suggested that some dinosaurs ventilated their lungs using either a hepatic piston mode of breathing, or a similar ‘visceral pump' driven by rotation of the pelvic bones [1,5]. However, there is no evidence that any of the fossil taxa analysed in this study possessed the associated smooth thoracic ceiling to facilitate the anterior–posterior translation of the viscera associated with the hepatic piston mechanism. All extinct taxa had low LD1 scores, and almost all of the species analysed had ‘forked' ribs associated with a furrowed thoracic ceiling and a dorsally immobile lung, which suggests that dinosaur taxa ventilated their respiratory system using costal aspiration [7,84] and perhaps a secondary mechanism associated with the gastralia or pelvic girdle [85,86]. Although some dinosaurs had their vertebral ribs fused to the vertebral column (e.g. *Ankylosaurus*) the major drivers of ventilation in extant archosaurs seem to be the sternal ribs, which would have remained mobile, and thus costal aspiration would still have been feasible [7,27].

Our findings have significant palaeobiological implications. The evolution of avian endothermy, and whether or not non-avian dinosaurs possessed similar levels of metabolism, has been a topic of considerable debate, and much of this has focused on whether the dinosaurian respiratory system was capable of sustaining such levels of metabolism [1–3]. Our results show dinosaurs had dorsally immobile, heterogeneously partitioned lungs, which could have supported high concentrations of gas-exchanging parenchyma [29] and potentially a thinned blood-gas barrier [5,87]. Recent studies have asserted that dinosaurs had high, or at least intermediate, levels of metabolism [88,89] and our results suggest that the dinosaurian respiratory system was capable of sustaining metabolic rates such as these. The dorsally immobile parabronchial lung structure of dinosaurs (and other archosaurs) would have been particularly advantageous in the Mesozoic, which is considered to have been a period of relative hypoxia compared with both modern day and with the preceding Permian [90,91], as the avian parabronchial lung is more efficient at extracting O_2_ under hypoxic conditions compared with the mammalian bronchoalveolar lung [92]. Although models of active competitive replacement of synapsids by dinosaurs have long been rejected [93], dinosaurs along with other archosaurs still probably had adaptations—the anatomy of the respiratory system being one [87,90,91]—which gave them an advantage in the aftermath of the end-Permian mass extinction [94].

## 5. Conclusion

In extant archosaurs, birds and crocodilians, the morphology of the dorsal vertebrae, and in particular that of the costovertebral joint, is very different and this reflects differences in the dorsal surfaces and structure of the adjacent lungs. Whereas crocodilians have a smooth thoracic ceiling and compliant lungs, birds possess a furrowed thoracic ceiling resulting in complete immobilization of the gas-exchanging regions of the lung. With this new analysis, we quantitatively show that all non-avian dinosaurs possessed costovertebral joints more similar in structure to birds than to crocodilians, and are reconstructed to have a dorsally immobilized, heterogeneously partition lung. The evolution of the dorsally immobilized lung ventilated by functionally decoupled air sacs, was a major evolutionary innovation along the avian stem. The relative volume constancy of the gas-exchanging portion of the avian lung is a prerequisite for the thinning of the blood-gas barrier, and extreme subdivision of the parabronchi. Similar pulmonary modifications would have provided dinosaurs with more efficient means of oxygen uptake relative to other vertebrates during the environmentally hypoxic conditions which pervaded much of the Mesozoic, thus potentially contributing to their radiation and dominance over terrestrial ecosystems.

## Supplementary Material

Supplementary Methods

## Supplementary Material

Supplementary Tables

## Supplementary Material

Supplementary Information

## Data accessibility

Landmarks, classifier data and the R code necessary to replicate the major findings of this analysis (the PCA, Procrustes ANOVA and LDA) are available in the electronic supplementary material of this paper.
