# Supplementary material for: Vertebral morphometrics and lung structure in non-avian dinosaurs
Source: R Soc Open Sci. 2018 Oct 24;5(10):180983. doi: 10.1098/rsos.180983 (PMC6227937; doi:10.1098/rsos.180983)
Supplement: Supplementary Tables [file rsos180983supp2.docx]

Royal Society Open Science

Vertebral Morphometrics and Lung Structure in Non-Avian Dinosaurs

Robert J. Brocklehurst, Emma R. Schachner, William I. Sellers

Supplementary Tables

Supplementary Table 1

Procrustes ANOVA table of main taxonomic groups included in this analysis

|  | Df | SSE | SS | R2 | F | Z | Pr(>F) |
| --- | --- | --- | --- | --- | --- | --- | --- |
| Y ~ Group | 360 | 16.156 | 14.472 | 0.47251 | 64.497 | 11.528 | 0.002 |

Effect sizes (Z)

|  | Aves | Crocodylomorpha | Dinosauromorpha | Ornithischia | Sauropoda | Theropoda |
| --- | --- | --- | --- | --- | --- | --- |
| Aves | 0 | 15.797 | 5.903 | 16.175 | 11.341 | 9.704 |
| Crocodylia | 15.797 | 0 | 10.618 | 16.571 | 14.22 | 14.01 |
| *Silesaurus* | 5.903 | 10.618 | 0 | 6.753 | 4.335 | 3.147 |
| Ornithischia | 16.175 | 16.571 | 6.753 | 0 | 5.485 | 4.454 |
| Sauropoda | 11.341 | 14.22 | 4.335 | 5.485 | 0 | 4.231 |
| Theropoda | 9.704 | 14.01 | 3.147 | 4.454 | 4.231 | 0 |

P-values

|  | Aves | Crocodylomorpha | Dinosauromorpha | Ornithischia | Sauropoda | Theropoda |
| --- | --- | --- | --- | --- | --- | --- |
| Aves | 1 | 0.002 | 0.002 | 0.002 | 0.002 | 0.002 |
| Crocodylia | 0.002 | 1 | 0.002 | 0.002 | 0.002 | 0.002 |
| *Silesaurus* | 0.002 | 0.002 | 1 | 0.002 | 0.002 | 0.01 |
| Ornithischia | 0.002 | 0.002 | 0.002 | 1 | 0.002 | 0.002 |
| Sauropoda | 0.002 | 0.002 | 0.002 | 0.002 | 1 | 0.006 |
| Theropoda | 0.002 | 0.002 | 0.01 | 0.002 | 0.006 | 1 |

Supplementary Table 2. Table of posterior probabilities for classification of dinosaur and dinosauriform vertebrae from the linear discriminant analysis.

| Genus | Vertebra | Bird_furrowed | Croc_furrowed | Croc_Smooth |
| --- | --- | --- | --- | --- |
| *Allosaurus* | 1 | 0.0222 | 0.9778 | 0 |
| *Allosaurus* | 2 | 0.9916 | 0.0084 | 0 |
| *Allosaurus* | 3 | 1 | 0 | 0 |
| *Allosaurus* | 4 | 1 | 0 | 0 |
| *Allosaurus* | 5 | 0.9998 | 0.0002 | 0 |
| *Allosaurus* | 6 | 0.9988 | 0.0012 | 0 |
| *Allosaurus* | 7 | 1 | 0 | 0 |
| *Allosaurus* | 8 | 0.9977 | 0.0023 | 0 |
| *Allosaurus* | 9 | 0.9989 | 0.0011 | 0 |
| *Allosaurus* | 10 | 0.9934 | 0.0066 | 0 |
| *Allosaurus* | 11 | 0.9995 | 0.0005 | 0 |
| *Allosaurus* | 12 | 0.9999 | 0.0001 | 0 |
| *Ankylosaurus* | 1 | 0.9996 | 0.0004 | 0 |
| *Ankylosaurus* | 2 | 0.9999 | 0.0001 | 0 |
| *Ankylosaurus* | 3 | 1 | 0 | 0 |
| *Ankylosaurus* | 4 | 1 | 0 | 0 |
| *Ankylosaurus* | 5 | 1 | 0 | 0 |
| *Ankylosaurus* | 6 | 1 | 0 | 0 |
| *Ankylosaurus* | 7 | 1 | 0 | 0 |
| *Ankylosaurus* | 8 | 1 | 0 | 0 |
| *Ankylosaurus* | 9 | 0.9998 | 0.0002 | 0 |
| *Ankylosaurus* | 10 | 0.9998 | 0.0002 | 0 |
| *Apatosaurus* | 1 | 1 | 0 | 0 |
| *Apatosaurus* | 2 | 1 | 0 | 0 |
| *Apatosaurus* | 3 | 1 | 0 | 0 |
| *Apatosaurus* | 4 | 1 | 0 | 0 |
| *Apatosaurus* | 5 | 1 | 0 | 0 |
| *Apatosaurus* | 6 | 1 | 0 | 0 |
| *Apatosaurus* | 7 | 1 | 0 | 0 |
| *Apatosaurus* | 8 | 1 | 0 | 0 |
| *Apatosaurus* | 9 | 1 | 0 | 0 |
| *Camarasaurus* | 1 | 0.9993 | 0.0007 | 0 |
| *Camarasaurus* | 2 | 0.9218 | 0.0782 | 0 |
| *Camarasaurus* | 3 | 1 | 0 | 0 |
| *Camarasaurus* | 4 | 0.0045 | 0 | 0.9955 |
| *Camarasaurus* | 5 | 1 | 0 | 0 |
| *Camarasaurus* | 6 | 1 | 0 | 0 |
| *Camarasaurus* | 7 | 1 | 0 | 0 |
| *Camarasaurus* | 8 | 1 | 0 | 0 |
| *Camarasaurus* | 9 | 1 | 0 | 0 |
| *Camarasaurus* | 10 | 0.021 | 0 | 0.979 |
| *Camarasaurus* | 11 | 1 | 0 | 0 |
| *Deinonychus* | 1 | 0.9988 | 0.0012 | 0 |
| *Deinonychus* | 4 | 1 | 0 | 0 |
| *Deinonychus* | 7 | 0.3668 | 0.6332 | 0 |
| *Deinonychus* | 10 | 0.9999 | 0.0001 | 0 |
| *Diplodocus* | 1 | 0.2978 | 0.7022 | 0 |
| *Diplodocus* | 2 | 0.9681 | 0.0319 | 0 |
| *Diplodocus* | 3 | 1 | 0 | 0 |
| *Diplodocus* | 4 | 1 | 0 | 0 |
| *Diplodocus* | 5 | 1 | 0 | 0 |
| *Diplodocus* | 6 | 1 | 0 | 0 |
| *Diplodocus* | 7 | 1 | 0 | 0 |
| *Diplodocus* | 8 | 1 | 0 | 0 |
| *Diplodocus* | 9 | 1 | 0 | 0 |
| *Diplodocus* | 10 | 1 | 0 | 0 |
| *Dryosaurus* | 1 | 0.9993 | 0.0007 | 0 |
| *Dryosaurus* | 2 | 0.9999 | 0.0001 | 0 |
| *Dryosaurus* | 3 | 0.995 | 0.005 | 0 |
| *Dryosaurus* | 4 | 0.9988 | 0.0012 | 0 |
| *Dryosaurus* | 5 | 0.9995 | 0.0005 | 0 |
| *Dryosaurus* | 6 | 0.9893 | 0.0063 | 0.0044 |
| *Dryosaurus* | 7 | 0.9924 | 0.0005 | 0.0071 |
| *Huayangosaurus* | ? | 1 | 0 | 0 |
| *Huayangosaurus* | ? | 1 | 0 | 0 |
| *Iguanodon* | 2 | 1 | 0 | 0 |
| *Iguanodon* | 4 | 1 | 0 | 0 |
| *Iguanodon* | 5 | 1 | 0 | 0 |
| *Iguanodon* | 9 | 0.9999 | 0.0001 | 0 |
| *Kritosaurus* | 2 | 0.9728 | 0.0272 | 0 |
| *Kritosaurus* | 3 | 1 | 0 | 0 |
| *Kritosaurus* | 4 | 1 | 0 | 0 |
| *Kritosaurus* | 8 | 1 | 0 | 0 |
| *Kritosaurus* | 9 | 0.9971 | 0.0029 | 0 |
| *Kritosaurus* | 10 | 0.9989 | 0.0011 | 0 |
| *Kritosaurus* | 11 | 0.9998 | 0.0002 | 0 |
| *Silesaurus* | 1 | 0.0008 | 0.9992 | 0 |
| *Silesaurus* | 2 | 0.0364 | 0.9636 | 0 |
| *Silesaurus* | 3 | 0.0001 | 0.9999 | 0 |
| *Silesaurus* | 4 | 0.1509 | 0.8491 | 0 |
| *Silesaurus* | 5 | 0.9364 | 0.0636 | 0 |
| *Silesaurus* | 6 | 0.9814 | 0.0186 | 0 |
| *Silesaurus* | 7 | 0.9998 | 0.0002 | 0 |
| *Silesaurus* | 8 | 0.9985 | 0.0015 | 0 |
| *Silesaurus* | 9 | 0.998 | 0.002 | 0 |
| *Silesaurus* | 10 | 0.9983 | 0.0017 | 0 |
| *Silesaurus* | 11 | 0.9999 | 0.0001 | 0 |
| *Silesaurus* | 12 | 0.6576 | 0.0075 | 0.3349 |
| *Silesaurus* | 13 | 0.9999 | 0.0001 | 0 |
| *Silesaurus* | 14 | 0.9996 | 0.0002 | 0.0002 |
| *Silesaurus* | 15 | 0.9999 | 0 | 0.0001 |
| *Silesaurus* | 16 | 1 | 0 | 0 |
| *Sinraptor* | 1 | 0.9958 | 0.0042 | 0 |
| *Sinraptor* | 2 | 0.9992 | 0.0008 | 0 |
| *Sinraptor* | 4 | 1 | 0 | 0 |
| *Sinraptor* | 9 | 1 | 0 | 0 |
| *Stegosaurus* | 1 | 0.9094 | 0.0906 | 0 |
| *Stegosaurus* | 2 | 0.9969 | 0.0031 | 0 |
| *Stegosaurus* | 3 | 0.9461 | 0.0539 | 0 |
| *Stegosaurus* | 4 | 0.9982 | 0.0018 | 0 |
| *Stegosaurus* | 5 | 1 | 0 | 0 |
| *Stegosaurus* | 6 | 0.9999 | 0.0001 | 0 |
| *Stegosaurus* | 7 | 0.9995 | 0.0004 | 0.0001 |
| *Stegosaurus* | 8 | 0.9972 | 0.0028 | 0 |
| *Stegosaurus* | 9 | 0.9999 | 0.0001 | 0 |
| *Stegosaurus* | 10 | 0.9999 | 0 | 0.0001 |
| *Stegosaurus* | 11 | 0.9996 | 0.0004 | 0 |
| *Stegosaurus* | 12 | 0.0023 | 0 | 0.9977 |
| *Stegosaurus* | 13 | 0.9725 | 0.0275 | 0 |
| *Styracosaurus* | 1 | 1 | 0 | 0 |
| *Styracosaurus* | 2 | 1 | 0 | 0 |
| *Styracosaurus* | 3 | 1 | 0 | 0 |
| *Styracosaurus* | 4 | 1 | 0 | 0 |
| *Styracosaurus* | 5 | 1 | 0 | 0 |
| *Styracosaurus* | 6 | 0.9999 | 0 | 0.0001 |
| *Styracosaurus* | 7 | 0.9993 | 0 | 0.0007 |
| *Styracosaurus* | 8 | 1 | 0 | 0 |
| *Styracosaurus* | 9 | 0.9976 | 0 | 0.0024 |
| *Styracosaurus* | 10 | 1 | 0 | 0 |
| *Styracosaurus* | 11 | 0.9865 | 0 | 0.0135 |
| *Styracosaurus* | 12 | 0.9778 | 0 | 0.0222 |
| *Triceratops* | 1 | 0.9999 | 0.0001 | 0 |
| *Triceratops* | 2 | 1 | 0 | 0 |
| *Triceratops* | 3 | 1 | 0 | 0 |
| *Triceratops* | 4 | 1 | 0 | 0 |
| *Triceratops* | 5 | 1 | 0 | 0 |
| *Triceratops* | 6 | 0.9981 | 0 | 0.0019 |
| *Triceratops* | 7 | 0.9989 | 0 | 0.0011 |
| *Triceratops* | 8 | 0.8907 | 0 | 0.1093 |
| *Triceratops* | 9 | 0.0043 | 0 | 0.9957 |
| *Triceratops* | 10 | 0.1335 | 0 | 0.8665 |
| *Triceratops* | 11 | 1 | 0 | 0 |
| *Triceratops* | 12 | 0.991 | 0.0001 | 0.0089 |
| *Triceratops* | 13 | 0.0002 | 0 | 0.9998 |
| *Triceratops* | 14 | 0.087 | 0 | 0.913 |
| *Tyrannosaurus* | 1 | 0.9998 | 0.0002 | 0 |
| *Tyrannosaurus* | 2 | 0.9969 | 0.0031 | 0 |
| *Tyrannosaurus* | 3 | 0.9996 | 0.0004 | 0 |
| *Tyrannosaurus* | 4 | 1 | 0 | 0 |
| *Tyrannosaurus* | 5 | 1 | 0 | 0 |
| *Tyrannosaurus* | 6 | 1 | 0 | 0 |
| *Tyrannosaurus* | 7 | 0.9971 | 0.0029 | 0 |
| *Tyrannosaurus* | 8 | 0.9993 | 0.0007 | 0 |
| *Tyrannosaurus* | 9 | 0.9982 | 0.0018 | 0 |
| *Tyrannosaurus* | 10 | 0.9999 | 0.0001 | 0 |
