# Supplementary material for: Vertebral morphometrics and lung structure in non-avian dinosaurs
Source: R Soc Open Sci. 2018 Oct 24;5(10):180983. doi: 10.1098/rsos.180983 (PMC6227937; doi:10.1098/rsos.180983)
Supplement: Supplementary Information [file rsos180983supp3.zip › Supplementary Info Proof/RSOS Supplementary Material Methods.docx]

Royal Society Open Science

Vertebral Morphometrics and Lung Structure in Non-Avian Dinosaurs

Robert J. Brocklehurst, Emma R. Schachner, William I. Sellers

Supplementary Methods

Supplementary Methods 1

Extant taxa used in this analysis

| **Class** | **Order** | **Family** | **Genus** | **Species** |
| --- | --- | --- | --- | --- |
| Reptilia | Crocodylia | Alligatoridae | *Alligator* | *mississipiensis* |
| Reptilia | Crocodylia | Alligatoridae | *Caiman* | *crocodilus* |
| Reptilia | Crocodylia | Crocodylidae | *Crocodylus* | *americanus* |
| Reptilia | Crocodylia | Gavialidae | *Gavialis* | *gangeticus* |
| Aves | Anseriformes | Anatidae | *Anser* | *anser* |
| Aves | Sphenisciformes | Spheniscidae | *Aptenodytes* | *patagonicus* |
| Aves | Apterygiformes | Apterygidae | *Apteryx* | *hastii* |
| Aves | Accipitriformes | Accipitridae | *Aquila* | *chrysaetos* |
| Aves | Pelecaniformes | Ardeidae | *Ardea* | *cinera* |
| Aves | Pelecaniformes | Balaenicipitidae | *Balaeniceps* | *rex* |
| Aves | Strigiformes | Strigidae | *Bubo* | *bubo* |
| Aves | Bucerotiformes | Bucerotidae | *Buceros* | *rhinoceros* |
| Aves | Bucerotiformes | Bucorvidae | *Bucorvus* | *abyssinicus* |
| Aves | Cacatuoidea | Cacatuidae | *Calyptorhynchus* | *baudini* |
| Aves | Cariamiformes | Cariamidae | *Cariama* | *cristata* |
| Aves | Cuculiformes | Cuculidae | *Centropus* | *sinenesis* |
| Aves | Anseriformes | Anhimidae | *Chauna* | *chavaria* |
| Aves | Ciconiiformes | Ciconiidae | *Ciconia* | *ciconia* |
| Aves | Anseriformes | Anatidae | *Coroscoba* | *coroscoba* |
| Aves | Procellariiformes | Diomedeidae | *Diomedea* | *epomophora* |
| Aves | Casuraiformes | Dromaidae | *Dromaius* | *novellhandiae* |
| Aves | Suliformes | Fregatidae | *Fregata* | *aquila* |
| Aves | Procellariiformes | Procellaridae | *Fulmarus* | *glacialis* |
| Aves | Charadriiformes | Haematopodidae | *Haematopus* | *ostralegus* |
| Aves | Otidiformes | Otididae | *Otis* | *tarda* |
| Aves | Accipitriformes | Accipitridae | *Pandion* | *haliaetus* |
| Aves | Podargiformes | Podargidae | *Podargus* | *strigoides* |
| Aves | Piciformes | Ramphastidae | *Ramphastos* | *tucanus* |
| Aves | Rheiformes | Rheidae | *Rhea* | *americana* |
| Aves | Accipitriformes | Sagittaridae | *Sagittarius* | *serpentarius* |
| Aves | Struthioniformes | Struthionidae | *Struthio* | *camelus* |
| Aves | Muscophagiformes | Musophagidae | *Tauraco* | *persa* |
| Aves | Charadriiformes | Laridae | *Thalasseus* | *sandvicensis* |

Supplementary Methods 2

Extinct taxa used in this analysis

| **Group** | **Genus** | **References and Specimen ID** |
| --- | --- | --- |
| Ornithischia | Ankylosaurus | Carpenter, 2004 |
| Sauropoda | Camarasaurus | AMNH 5760, 5761, Osborn and Mook, 1921 |
| Dinosauromorpha | Silesaurus | Piechowski and Dzik, 2010 |
| Ornithischia | Huayangosaurus | Maidment et al., 2006 |
| Ornithischia | Iguanodon | Hooley, 1925; Norman, 1980 |
| Ornithischia | Kritosaurus | Lull and Wright, 1942 |
| Ornithischia | Dryosaurus | CM 3392, Galton, 1981 |
| Sauropoda | Apatosaurus | Gilmore, 1936 |
| Sauropoda | Diplodocus | Hatcher, 1901 |
| Ornithischia | Stegosaurus | Maidment et al., 2015 |
| Ornithischia | Styracosaurus | Holmes et al., 2005 |
| Theropoda | Allosaurus | Madsen Jr, 1976 |
| Theropoda | Deinonychus | YPM 5210, Ostrom, 1969 |
| Theropoda | Sinraptor | Currie and Zhao, 1993 |
| Theropoda | Tyrannosaurus | Brochu, 2003 |
| Ornithischia | Triceratops | Ostrom and Wellnhofer, 1986 |

Supplementary Methods 3

Landmark definitions used in the geometric morphometric analysis.

| Landmark | Description |
| --- | --- |
| 1 | Midline dorsal margin of the centrum |
| 2 | Lateral-most point of the centrum |
| 3 | Midline ventral margin of the centrum |
| 4 | Midline ventral margin of the neural arch |
| 5 | Midline dorsal margin of the neural arch |
| 6 | Dorsal margin of the neural spine |
| 7 | Ventromedial margin of the prezygapohyseal articular facet |
| 8 | Dorsolateral margin of the prezygapohyseal articular facet |
| 9 | Lateral contact between the neural arch and prezygapophyseal body |
| 10 | Ventromedial edge of the parapophysis/ventrolateral edge of lumbar transverse process |
| 11 | Dorsomedial edge of the diapophysis/dorsolateral edge of lumbar transverse process |

**Supplementary References**

Brochu, C.A., 2003. Osteology of Tyrannosaurus rex: insights from a nearly complete skeleton and high-resolution computed tomographic analysis of the skull. J. Vertebr. Paleontol. 22, 1–138.

Carpenter, K., 2004. Redescription of *Ankylosaurus magniventris* Brown 1908 (Ankylosauridae) from the Upper Cretaceous of the Western Interior of North America. Can. J. Earth Sci. 41, 961–986. https://doi.org/10.1139/e04-043

Currie, P.J., Zhao, X.-J., 1993. A new carnosaur (Dinosauria, Theropoda) from the Jurassic of Xinjiang, People’s Republic of China. Can. J. Earth Sci. 30, 2037–2081.

Galton, P.M., 1981. Dryosaurus, a hypsilophodontid dinosaur from the upper jurassic of north America and Africa postcranial skeleton. Paläontol. Z. 55, 271–312.

Gilmore, C.W., 1936. Osteology of Apatosaurus, with Species Reference to Specimens in the Carnegie Museum. Mem. Carnegie Mus. 11, 175–300.

Hatcher, J., 1901. Diplodocus (Marsh): its osteology, taxonomy, and probable habits, with a restoration of the skeleton. Mem. Carnegie Mus. 1, 1–64.

Holmes, R.B., Ryan, M.J., Murray, A.M., 2005. Photographic atlas of the postcranial skeleton of the type specimen of Styracosaurus albertensis with additional isolated cranial elements from Alberta. Syllogeus 75, 1–75.

Hooley, R.W., 1925. On the skeleton of Iguanodon atherfieldensis sp. nov., from the Wealden Shales of Atherfield (Isle of Wight). Q. J. Geol. Soc. 81, 1–61.

Lull, R.S., Wright, N.E., 1942. Hadrosaurian dinosaurs of North America. Geol. Soc. Am. Spec. Pap. 40, 1–272.

Madsen Jr, J.H., 1976. Allosaurus fragilis: a revised osteology. Utah Geol. Min. Surv. Bull. 109, 1–163.

Maidment, S.C., Wei, G., Norman, D.B., 2006. Re-description of the postcranial skeleton of the Middle Jurassic stegosaur Huayangosaurus taibaii. J. Vertebr. Paleontol. 26, 944–956.

Maidment, S.C.R., Brassey, C., Barrett, P.M., 2015. The Postcranial Skeleton of an Exceptionally Complete Individual of the Plated Dinosaur Stegosaurus stenops (Dinosauria: Thyreophora) from the Upper Jurassic Morrison Formation of Wyoming, U.S.A. PLOS ONE 10, e0138352. https://doi.org/10.1371/journal.pone.0138352

Norman, D.B., 1980. On the ornithischian dinosaur Iguanodon bernissartensis from the lower Lower Cretaceous of Bernissart (Belgium). Mem. Inst. R. Sci. Nat. Belg. 178, 1–105.

Osborn, H.F., Mook, C.C., 1921. Camarsaurus, Amphicoelias and other sauropods of Cope. Mem. Am. Mus. Nat. Hist. 3, 247–440.

Ostrom, J.H., 1969. Osteology of Deinonychus antirrhopus, an unusual theropod from the Lower Cretaceous of Montana. Bull. Peabody Mus. Nat. Hist. 30, 1–165.

Ostrom, J.H., Wellnhofer, P., 1986. The Munich specimen of Triceratops with a revision of the genus. Zitteliana 11, 111–158.

Piechowski, R., Dzik, J., 2010. The axial skeleton of Silesaurus opolensis. J. Vertebr. Paleontol. 30, 1127–1141.
